# Relationships between parental factors and child anxiety and depressive symptoms in an indicated preventive intervention

**DOI:** 10.1007/s00787-025-02831-5

**Published:** 2025-08-18

**Authors:** Kristin Ytreland, Elisabeth Valmyr Bania, Carina Lisøy, Stian Lydersen, Simon-Peter Neumer, Frode Adolfsen, Kristin Dagmar Martinsen, Anne Mari Sund, Jo Magne Ingul

**Affiliations:** 1 Regional Centre for Child and Youth Mental Health and Child Welfare - Central Norway, Department of Mental Health, Faculty of Medicine and Health Sciences, Norwegian University of Science and Technology (NTNU), Trondheim, Norway; 2 Regional Centre for Child and Adolescent Mental Health - Eastern and Southern Norway, Oslo, Norway; 3 Regional Centre for Child and Youth Mental Health and Child Welfare – Northern Norway, Faculty of Health Sciences, UIT, The Arctic University of Norway, Tromsø, Norway; 4 Department of Psychology, University of Oslo, Oslo, Norway; 5 St. Olavs Hospital HF, PH - Avd. for barne- og ungdomspsykiatri, Trondheim, Norway

**Keywords:** Anxiety, Depression, Parenting, Family functioning, Prevention, Children

## Abstract

**Background:**

Parental factors such as parental symptomatology, family functioning, and parental practices are linked to child anxiety and depression. While most research focuses on clinical groups, this study examines these associations in an indicated sample, before and after a preventive intervention and at 12-month follow-up.

**Methods:**

Participants included 1043 parents of 650 children (8–12 years old) from the ECHO trial, aimed at optimizing a preventive program for anxious and sad children. Using cross-lagged panel models, we modelled the relationship between parent-reported children’s anxiety and depression symptoms and parental factors. Parental factors were parents’ symptoms of anxiety and depression, family functioning and parental practices (managing emotions, setting goals & dealing with problems, and dealing with negative emotions). All variables were measured by parent-report questionnaires at baseline, post-intervention and 12-month follow-up.

**Results:**

Positive associations were found between child anxiety and depressive symptoms, and parental anxiety and depressive symptoms and poorer family functioning. There were smaller, negative associations between children’s symptoms and positive parental practices.

**Conclusion:**

Parental factors were significantly associated with child anxiety and depression over time, with stronger links to parents’ anxiety and depressive symptoms and family functioning, than parental practices.

## Introduction

Schleider and Weisz [1] propose a theoretical model highlighting the complex interplay between family dynamics and child internalizing problems. This triadic model proposes modifiable processes on three levels within the family: *parent-level*, e.g. parent symptomatology; *family-level*, e.g. family functioning; and *dyad-level*, e.g. parental practices. These processes may affect the child’s cognitive style and attentional biases, thereby influencing the development of internalizing problems, such as anxiety or depression. The model also suggests that changes in children’s internalizing problems can impact processes on all three levels within the family. If maladaptive family processes may lead to higher child symptom levels, which can lead to even less adaptive family processes, addressing factors at the parent, family, and dyad levels can potentially create positive ripple effects that improve both child, parent, and family well-being [1].

At the *parent-level*, numerous studies have shown that parents’ and children’s anxious and depressive problems are linked to each other. In a meta-analysis, Lawrence et al. [2] found that offspring of parents with anxiety disorders were at 76% higher risk of developing anxiety, and 31% higher risk of developing depression, compared to parents without an anxiety disorder. In a longitudinal study spanning 25 years and three generations, Jacobs et al. [3] found that parents’ depression increased the risks of offspring anxiety and depression. Offspring of mothers with major depressive disorder (MDD) had a 130% higher risk of lifetime anxiety, and 110% higher risk of MDD compared to offspring of mothers without MDD. Similarly, offspring of fathers with MDD had a 90% higher risk of lifetime anxiety and a 60% higher risk of lifetime MDD compared to offspring of fathers with no MDD. Furthermore, maternal MDD was associated with MDD onset in childhood, while paternal MDD was associated with anxiety onset in adulthood [3]. Eckshtain et al. [4] found that parents´ depressive symptoms at baseline negatively predicted child treatment response. For children who received treatment for internalizing problems, children whose parents had elevated depression scores showed an increase in internalizing symptoms over time, while children of parents without elevated depression showed a decline. This pattern was more prominent in parent-reports [4].

At the *family-level*, family functioning is also related to the development and maintenance of child anxiety and depression. Family functioning refers to how family members interact, cooperate, and support one another to achieve goals and address challenges [5]. It encompasses aspects such as communication, emotional connections, roles, adaptability, and problem-solving. Poorer family functioning is associated with increased symptoms of anxiety and depression, as well as lower overall functioning in children and adolescents [6, 7]. In an RCT with 230 children treated for anxiety disorders, poorer parent-reported family functioning was significantly associated with elevated anxiety symptoms and disorder severity, as well as poorer child functioning reported by children, parents and clinicians [6]. Both mothers and fathers of children with anxiety disorders reported poorer family functioning, compared to parents of children without mental disorders. However, only mothers´ reported family functioning was significantly correlated with children’s depressive symptoms. Parents’ symptoms of anxiety and depression also predicted poorer family functioning [6]. In a universal preventive program, Kennedy et al. [7] found that child-reported family functioning predicted child internalizing symptoms six months post intervention.

On the *parent-child dyad-level*, parental practices are linked to child anxiety and depressive symptoms. Based on a meta-analysis and systematic review by Yap and Jorm [8], and a consensus of international experts [9], parental guidelines were developed to prevent child anxiety and depression [10]. Based on these guidelines, the *Parenting Resilient Kids* (PaRK) program aims to improve parental risk and protective factors for child anxiety and depression [11]. Participants complete a baseline survey, including the Parenting to Reduce Child Anxiety and Depression Scale (PaRCADS) [12], which measures parents’ adherence to the aforementioned guidelines [10]. *PaRK* provide parents with individual feedback and up to twelve interactive modules based on their baseline assessment, and in an effectiveness trial of *PaRK*, the control group received general educational factsheets [11]. Both intervention and control groups showed improvements in children’s symptoms and family functioning, with no statistically significant group difference. However, parents in the intervention group reported greater improvements in PaRCADS-score compared to the control group. At the 12-month follow-up, there were no mediation effects on children´s symptoms based on post-intervention PaRCADS-scores [11]. In the current study, we included three parental practices measured by the PaRCADS: *Managing emotions* (emotion regulation and resilience); *Setting goals & dealing with problems* (goal setting, problem-solving and perseverance); and *Dealing with negative emotions* (helping their child manage and cope with their anxious or sad emotions before they become a problem).

Most studies that have investigated the associations between parental symptomatology or family functioning and children’s anxiety and depressive symptoms, focus on clinical samples, whereas we examine an indicated sample who received a preventive intervention. This study is part of the ECHO trial [13], which aimed to optimize the preventive program *Emotion* [14] for 8-12-year-olds who are more anxious and/or sad than their peers. To the best of our knowledge, no research has examined the associations between individual PaRCADS domain scores and children’s symptoms over time. Based on previous research, we hypothesize positive correlations between parent-reported child symptoms and parents’ symptoms of anxiety and depression and poorer family functioning. We also hypothesize negative correlations between parent-reported child symptoms and positive parental practices targeted in the parental component of the *Emotion* intervention [15]: *managing emotions*, *setting goals & dealing with problems*, and *dealing with negative emotions*, within the same measurement time. Lastly, we hypothesize that parental factors at baseline and post-intervention predict child anxiety and depressive symptoms one measurement time later (post-intervention and 12-month follow-up).

## Methods

The current study used data obtained in a randomized factorial trial, ECHO (clinicaltrials.gov NCT042633558). The trial was approved by the Regional Committees for Medical and Health Research Ethics (REK)– Southeast Norway (ref. 2019/1198), and the Norwegian Centre for Research Data (ref. 152745). The ECHO trial employed a 2 × 2 × 2 (2^3^) factorial design to optimize a preventive intervention for child anxiety and depression. We present details about the trial relevant to this study below, the interested reader may find more details about the project design in the protocol article [13], and the rationale and proposed mechanisms behind the factorial trial are described in Ingul et al. [16].

### Procedure

*Emotion* is a transdiagnostic, indicated preventive, manual-based intervention for children 8–12 years old, completed over eight to ten weeks [14]. In the randomized ECHO trial, children participated in 16 face-to-face child sessions, or eight sessions face-to-face and eight alternating sessions delivered automated online. Parents participated in five parent group sessions or received a psychoeducational brochure.

Post-intervention outcomes from the ECHO-trial showed a general decline in children´s symptoms across all conditions, with no statistically significant differences between intervention levels [17]. This suggests that participants on average reported reduced anxiety and depression symptoms, regardless of experimental condition, for example whether parents received five parent group sessions or a brochure. Additionally, there were no statistically significant differences in parental factors related to child emotional problems (parent’s symptoms, family functioning, parenting styles or parental practices) between parents in the group sessions condition and in the brochure condition [18]. Therefore, the current study draws data from the total sample of parents in the trial, regardless of experimental condition.

### Participants

**Fig. 1:**
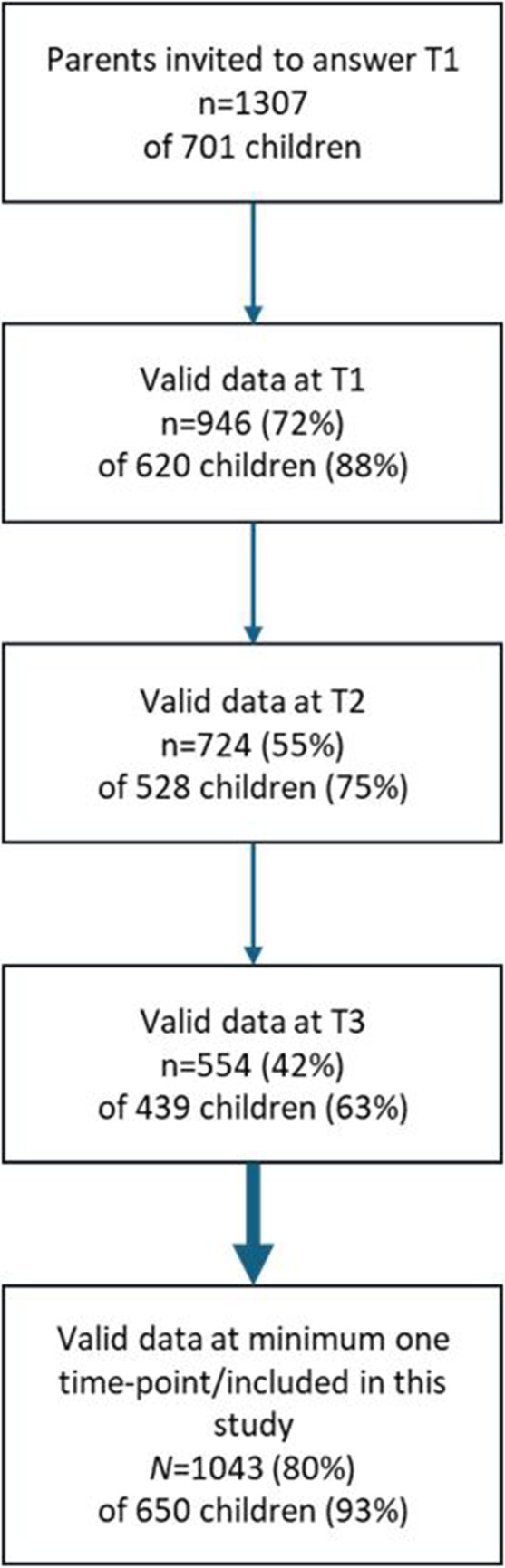
Participant flow

Children, 8–12 years old, and their parents were recruited in 58 public schools across Norway, in five waves of data collection. Self-report versions of The Multidimensional Anxiety Scale for Children, MASC-C [19] and the Short Moods and Feelings Questionnaire, SMFQ-C [20], was administered to 1315 children. Children with symptom scores of ≥ one standard deviation above means previously reported in population-based samples in either instrument, were eligible for the study. Cut-off values for MASC-C were: girls ≥ 61 points; MASC-C boys ≥ 54 points, based on Olason et al. [21], and for SMFQ-C for all participants: ≥ 7 points, based on Rhew et al. [22]. These results determined their inclusion in the intervention and are not included in the current study. Up to seven children per school, per wave, were invited to attend *Emotion* groups. Parents (*n* = 1307) of the enrolled children (*n* = 701) were invited to answer an online questionnaire. Participant flow is illustrated in Fig. 1.

### Measures

Children´s age was collected in the consent form, while other demographic information was collected the first time parents answered our survey, at baseline (T1), post-intervention (T2), or 12-month-follow-up (T3). Parents also responded to several questionnaires, those used in the current trial are described below.

#### Parent´s symptoms of anxiety and depression

The Hopkins Symptoms Checklist - HSCL-10 [23] is a 10-item self-report scale that measures psychological distress, mainly anxiety and depressive symptoms. Items are rated on a 4-point scale from 0 (*not at all*) to 3 (*extremely*) and aggregate into a total sum score, with higher scores indicating higher symptom levels. A previous study [24] reported good internal consistency in a large Norwegian sample, comparable to our sample, where McDonald´s Omega was ω = 0.88.

#### Family functioning

The Family Assessment Device, General Functioning subscale - FAD-GF [5], is a 12-item self-report scale measuring families’ healthy and unhealthy functioning. Responses range from 1 (*strongly agree*) to 4 (*strongly disagree*). Negatively worded statements were reverse-coded, so that higher total scores consistently indicate poorer family functioning. Internal consistency was good in a previous Norwegian study [25], similar to our sample, ω = 86.

#### Parental practices

The Norwegian version of the Parenting to Reduce Child Anxiety and Depression Scale - PaRCADS(N) [12, 25], consists of 78 items across ten domains. This study used three domains to measure three parental practices targeted in the *Emotion* intervention: *Managing emotions*, which focus on emotion regulation and resilience; *Setting goals & dealing with problems*, which focus on goal setting, problem-solving and perseverance; and *Dealing with negative emotions*, which focus on helping children manage and cope with their anxious or sad emotions before they become a problem.

Items are rated on a 5-point scale from 0 (*almost never*) to 4 (*almost always*), or a likelihood scale for hypothetical questions from 0 (*very unlikely*) to 4 (*very likely*). Negatively worded items are reverse-coded, and higher scores indicate more adaptive parental practices. PaRCADS(N) showed below adequate to adequate psychometric properties in a Norwegian population sample [25]. Internal consistency values were similar in this study for the three domains: *Managing emotions* (ω = 0.54), *Setting goals & dealing with problems* (ω = 0.71), and *Dealing with negative emotions* (ω.63).

#### Children’s symptoms

The Multidimensional Anxiety Scale for Children, parent-report - MASC-P [19], measures anxiety symptoms in 8–19-year-olds with 39 items rated on a 4-point scale from 1 (*never*) to 4 (*often*). The Norwegian version has shown good internal consistency, and construct validity [26]. The short form of the Mood and Feelings Questionnaire, parent-report, SMFQ-P [20], measures depressive symptoms in 6-18-year-olds with 13 items rated on a 3-point scale from 0 (*not true*) to 2 (*true*). For both symptom scales, the total score is aggregated from the sum of all item responses and higher scores indicate higher symptom levels. In the original RCT of *Emotion* [27], internal consistency of MASC-P and SMFQ-P was excellent, as in the current sample, ω = 0.90 for both scales.

### Statistical analyses

To explore the relationships between parental factors and child anxiety and depression symptoms before, after, and 12 months following a preventive school-based intervention, we used five separate cross-lagged panel models. Each model included one of the five parental factors: *parents´ symptoms of anxiety and depression*,* family functioning*,* managing emotions*,* setting goals & dealing with problems*, and *dealing with negative emotions*, alongside parent-reported child anxiety and depression symptoms over the three measurement times. These models enabled us to assess whether a parental factor at T1 (or T2) predicted child symptoms at T2 (or T3) while controlling for the children’s symptoms at baseline.

Concurrent associations were included to assess the relationship between a parental factor and child symptoms within the same time. We examined effects over time using cross-lagged paths, accounting for lag-1 effects, the impact of a parental factor on child symptoms on the immediately following measurement time, i.e. from parental factor at T1 (or T2) to child symptoms at T2 (or T3). To model the stability of parental factors and child symptoms across time, we included first-order autoregressive paths, i.e. linking a variable at baseline with itself post-intervention and post-intervention with 12-month follow-up. To establish adequate model fit, we added second-order autoregressive effects, i.e. linked a variable at T1 with itself at T3. Additionally, we allowed residual correlations between child symptoms of depression and anxiety within the same time points, i.e. linking anxiety with depression at the same measurement time.

We used maximum likelihood estimation with robust standard errors (MLR), which estimates parameters for participants with partly missing data using full information maximum likelihood. Our sample contained up to two parents for the same child, thus, child-ID was used as a cluster variable to obtain more accurate estimates, accounting for non-independence of the data. Due to large variability in scale size between our continuous variables, we report standardized coefficients (STDYX) [28]. Model fit assessment relies on a combination of indices, such as the chi-square (χ2) test, comparative fit index (CFI), Tucker-Lewis index (TLI), and the Root Mean Square Error of Approximation (RMSEA) with its corresponding 90% confidence interval (CI). We considered RMSEA values below 0.06, and CFI and TLI values above 0.95, as indicative of relatively good model fit [29]. In separate explorative analyses, we adjusted for the effects of child symptoms on later parental factors, in addition to adding lag-2 effects, i.e. the effect with a time lag of two measurement times, i.e. from baseline to 12-month follow-up. The results were substantially unchanged; data are not shown. Panel models were estimated in Mplus version 8 [28].

## Results

This study included 1043 parents of 650 children, with survey data from at least one measurement time. Ten participants were foster parents, while two were stepparents for the enrolled child. Parents’ enrolled children were between 8.6 and 12.8 years old (*M* = 10.5, *SD* = 0.7), 61% were parents of girls. 80% of parents were full-time employed. Of those who were not currently working (9%), the most frequent reasons were “unable to work” (3%) and “under work assessment allowance” (2%). 66% of parents attained education at university or college, which is a larger proportion than the same age group in the Norwegian population (50%) [30]. 43% (*n* = 446) were in the parent group session condition.

This indicated sample deviates somewhat from previous population samples, with higher parent-reported symptom scores for both parents and their children. Converting the HSCL-10 mean item scores from Strand et al. [24] for comparison, they found a mean total score on parental psychopathology of 4.3 among 25–44-year-olds. Parent-reported child depression symptoms (SMFQ-P) were higher in our sample compared to the PaRCADS(N) validation study (*M* = 4.0, *SD* = 4.3). In an Australian community sample of children aged 8–13 [31], parent-reported anxiety symptoms (MASC-P) were 41.1 (*SD* = 13.5) for boys and 47.9 (*SD* = 14.8) for girls. FAD-GF and PaRCADS(N) scores were slightly poorer, but comparable to the community-based sample of parents of 8-12-year-olds in the PaRCADS(N) validation study [25]. Further descriptive information is reported in Table 1.

**Table 1:** Descriptive statistics, N=1043, unless otherwise specified

Parent characteristics		*n*	%
Age in years, *M*(*SD*)		41.9 (6.1)	
**Sex** (*n* = 1039)			
Female		628	60%
Male		411	40%
**Completed education**			
Lower secondary school		170	16%
Upper secondary school		182	17%
University/College ≤ 4 years		336	32%
University/College > 4 years		355	34%
**Parents evaluation of family income**			
Very good		135	13%
Good		619	59%
Mediocre		264	25%
Poor		20	2%
Very poor		5	1%
**Employment**			
Full time		837	80%
Part time		117	11%
Currently not working		89	9%

Measurement time ***n*** (range)	Baseline 921–946	Post-intervention 673–724	12-month follow-up 531–554
	***M*** **(** ***SD*** **)**	***M*** **(** ***SD*** **)**	***M*** **(** ***SD*** **)**
**Parental factors**			
Parent symtoms (HSCL-10)	4.9 (4.6)	4.6 (4.7)	4.5 (4.4)
Family functioning (FAD-GF)	19.3 (4.7)	19.4 (4.6)	19.7 (4.7)
Managing emotions	19.3 (3.4)	20.1 (3.3)	20.1 (3.3)
Setting goals & dealing with problems	23.7 (3.9)	24.3 (3.6)	23.9 (3.7)
Dealing with negative emotions	28.4 (3.8)	29.4 (3.7)	29.2 (4.0)
**Children´s symptoms**			
Anxiety (MASC-P)	51.8 (15.4)	48.8 (15.1)	46.5 (16.5)
Depression (SMFQ-P)	6.8 (5.0)	5.7 (5.0)	5.5 (5.2)

*HSCL-10 *Hopkins Symptoms Checklist − 10 item version,* FAD-GF *Family Assessment Device - General Functioning subscale, *MASC-P* The Multidimensional Anxiety Scale for Children - Parent-report,* SMFQ-P *the short form of the Mood and Feelings Questionnaire - Parent-report*.*

### Cross-lagged panel models

**Fig. 2:**
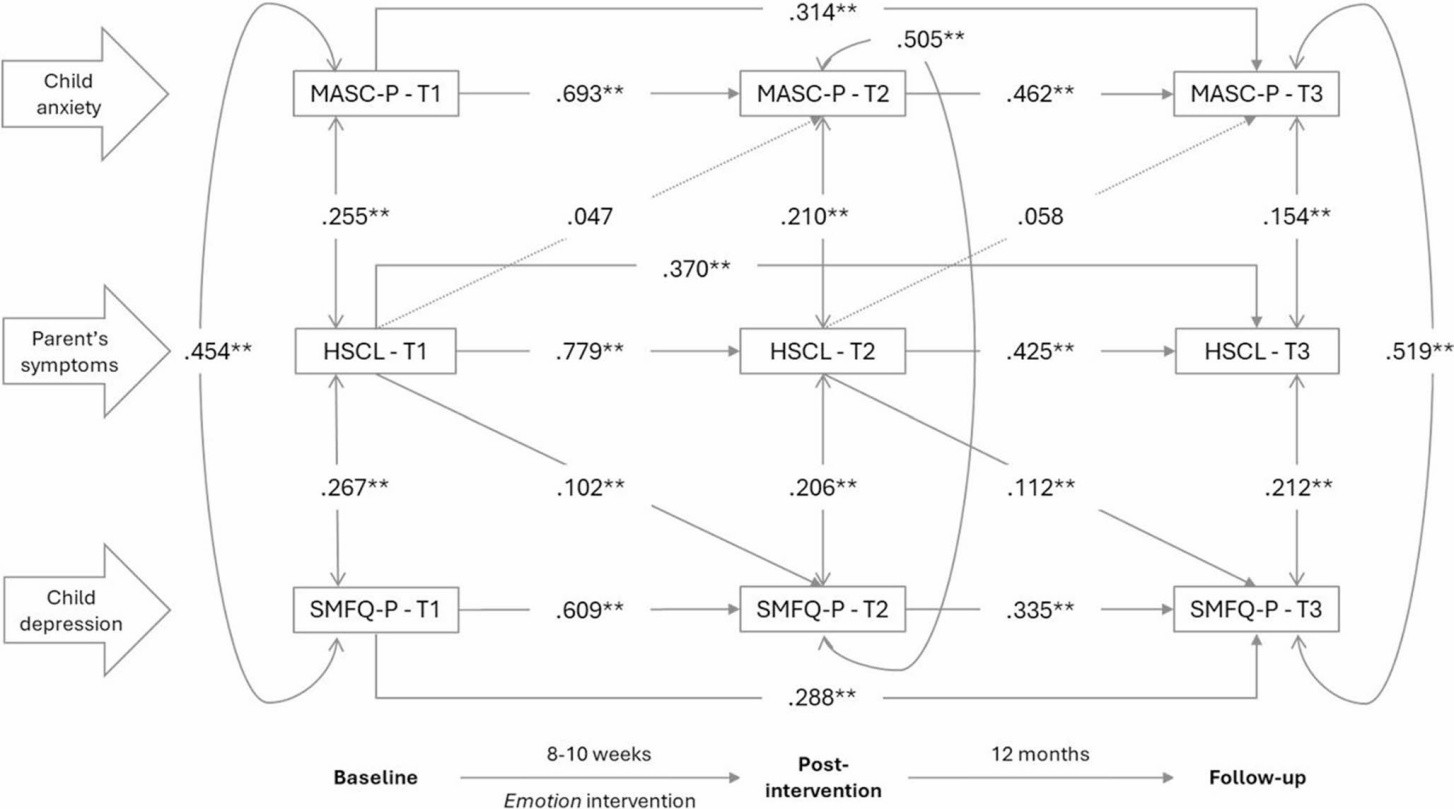
Associations, given by standardized coefficients (STDYX) between parent anxiety and depression symptoms (HSCL-10) and parent-reported child anxiety (MASC-P) and depression (SMFQ-P) at baseline (T1), post-intervention (T2) and 12-month follow-up (T3). Model fit indices: RMSEA = 0.021 (90% C.I.=0.00 to 0.04), CFI = 0.997, TLI = 0.993. * *p* ≤.05; **p ≤.01

**Fig. 3:**
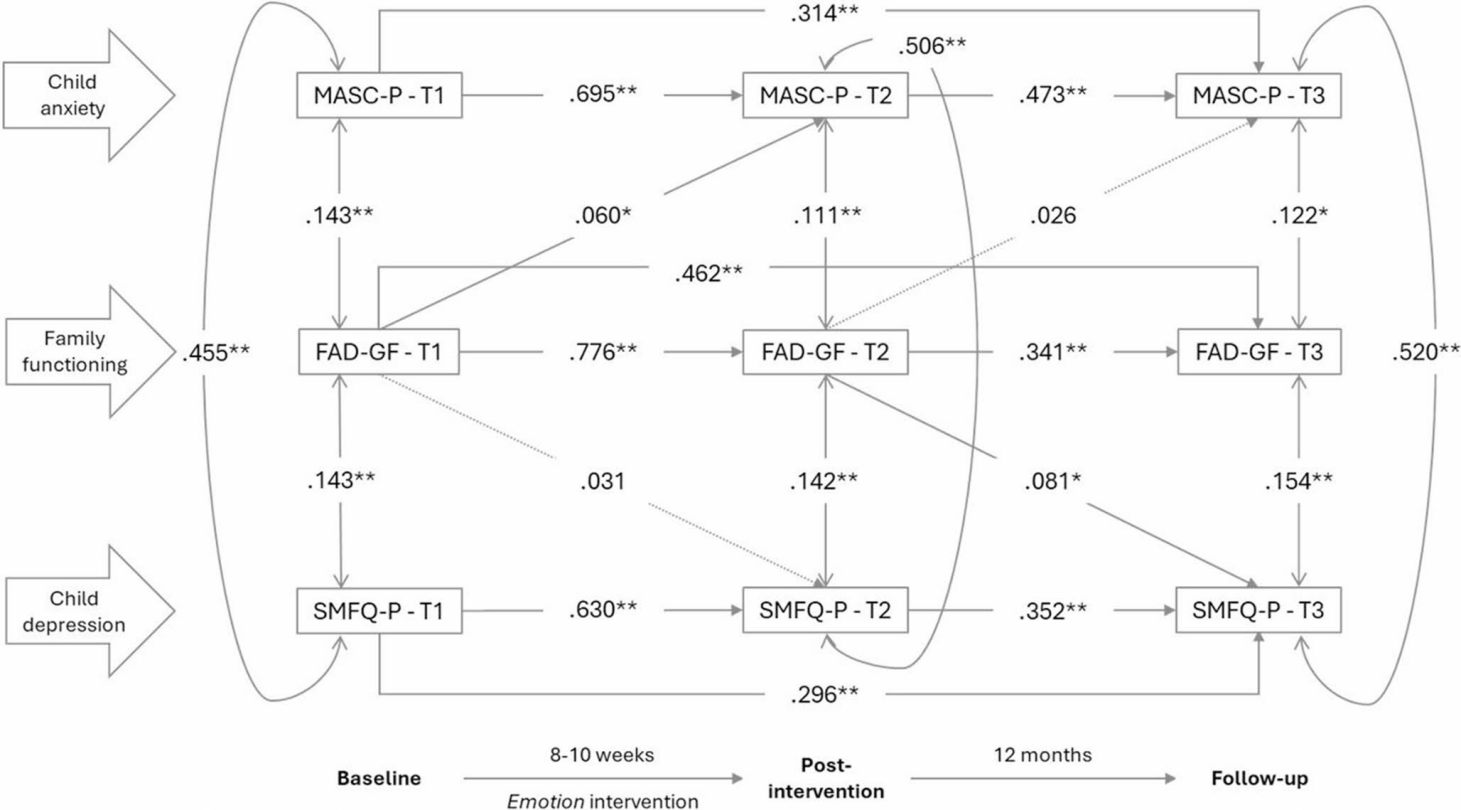
Associations, given by standardized coefficients (STDYX) between family functioning (FAD-GF) and parent-reported child anxiety (MASC-P) and depression (SMFQ-P) at baseline (T1), post-intervention (T2) and 12-month follow-up (T3). Model fit indices: RMSEA = 0.022 (90% C.I.=0.00 to 0.04), CFI = 0.997, TLI = 0.993. * *p* ≤.05; **p ≤.01

**Fig. 4:**
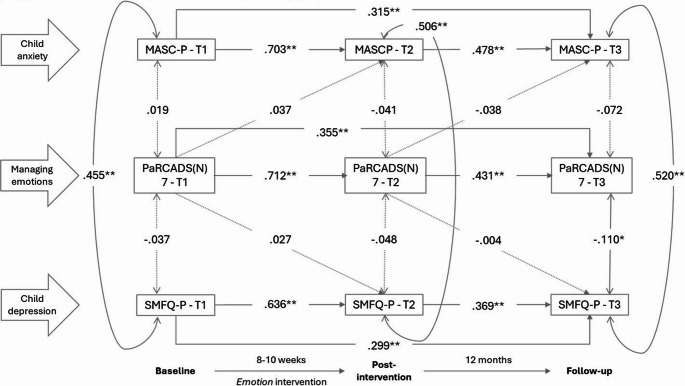
Associations, given by standardized coefficients (STDYX) between the parental practice managing emotions (PaRCADS(N) 7) and parent-reported child anxiety (MASC-P) and depression (SMFQ-P) at baseline (T1), post-intervention (T2) and 12-month follow-up (T3). Model fit indices: RMSEA = 0.024 (90% C.I.=0.00 to 0.04), CFI = 0.996, TLI = 0.991. * *p* ≤.05; **p ≤.01.

**Fig. 5:**
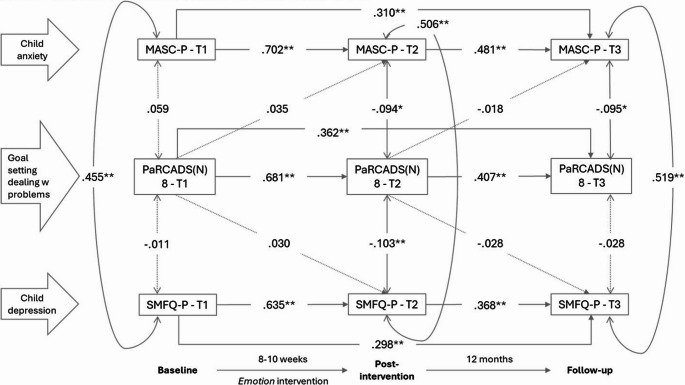
Associations, given by standardized coefficients (STDYX) between the parental practice setting goals & dealing with problems (PaRCADS(N) 8) and parent-reported child anxiety (MASC-P) and depression (SMFQ-P) at baseline (T1), post-intervention (T2) and 12-month follow-up (T3). Model fit indices: RMSEA = 0.023 (90% C.I.=0.00 to 0.04), CFI = 0.996, TLI = 0.991. * *p* ≤.05; **p ≤.01.

**Fig. 6:**
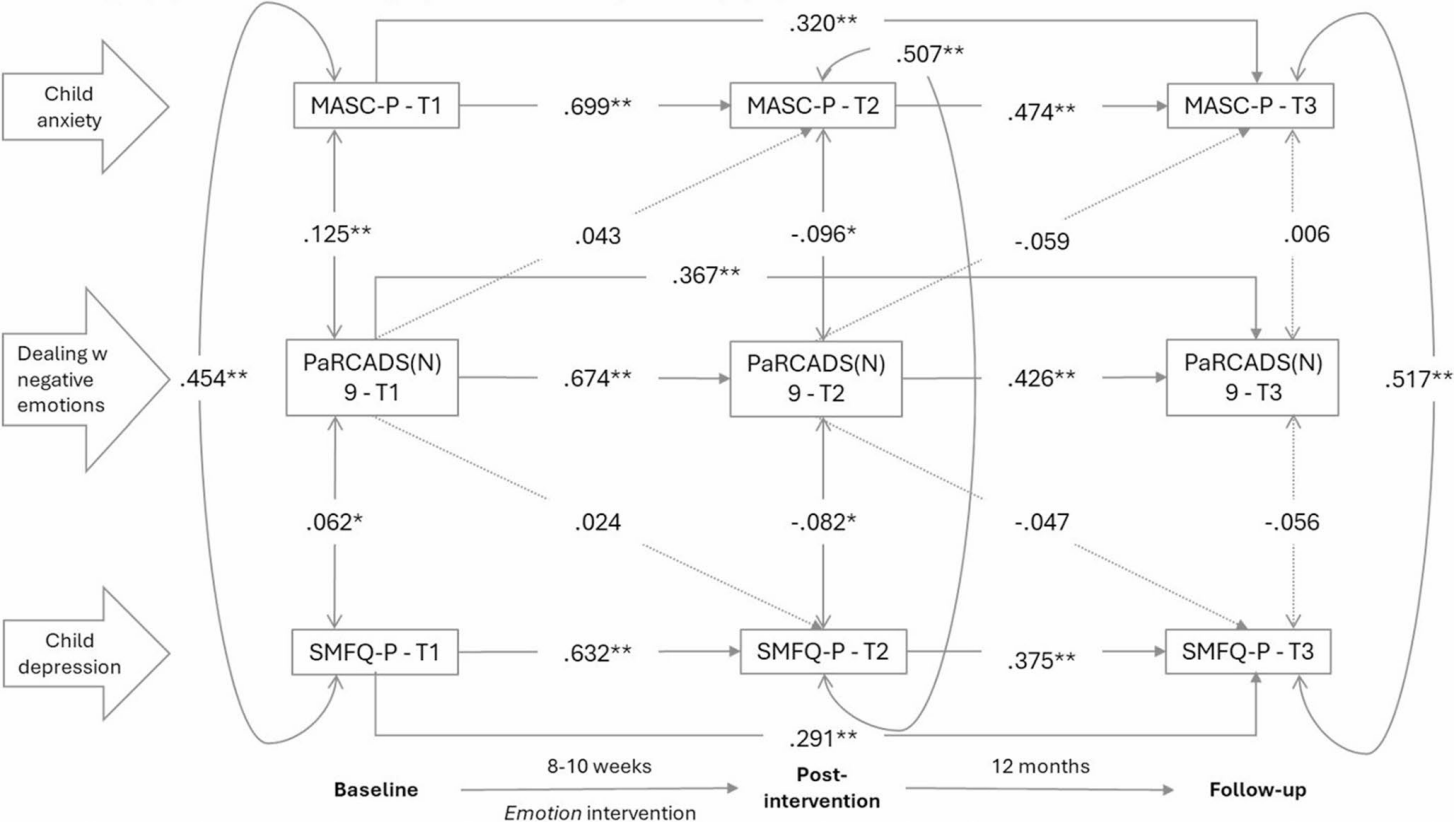
Associations, given by standardized coefficients (STDYX) between the parental practice dealing with negative emotions (PaRCADS(N) 9) and parent-reported child anxiety (MASC-P) and depression (SMFQ-P) at baseline (T1), post-intervention (T2) and 12-month follow-up (T3). Model fit indices: RMSEA = 0.027 (90% C.I.=0.01 to 0.04, CFI = 0.995, TLI = 0.988. * *p* ≤.05; **p ≤.01

Model fit was relatively good for all five models, with RMSEA ranging from 0.021 to 0.027, CFI from 0.995 to 0.997 and TLI from 0.988 to 0.993. Model results are presented in Figs. 2 to 6. All variables significantly predicted itself at the later measurement times (autoregressive effects). From T1 to T2, estimates ranged from 0.609 to 0.779; from T2 to T3, estimates ranged from 0.335 to 0.481; from T1 to T3, estimates ranged from 0.111 to 0.370. There were statistically significant positive correlations between parent-reported anxiety symptoms (MASC-P) and depressive symptoms (SMFQ-P) within each timepoint, with estimates ranging from 0.454 to 0.520. 

### Parental symptomatology

Figure 2 shows the associations between parents’ anxiety and depressive symptoms and child anxiety and depressive symptoms. There were statistically significant correlations between parents´ and children’s symptoms within all three measurement times ranging from.267 for depression at T1 to.154 for anxiety at T3. There were positive, statistically significant cross-lagged paths from parents’ symptoms at T1 to child depression at T2 (.102), and from T2 to T3 (.112). That is, higher parent symptoms predicted higher child depressive symptoms at the later measurement time.

## Family functioning

Figure 3 shows the associations between family functioning and child anxiety and depressive symptoms. Statistically significant correlations were found at all three times, with poorer family functioning associated with higher child symptoms. Positive cross-lagged paths were observed from family functioning at T1 to child anxiety at T2 and from family functioning at T2 to depression at T3. Hence, poorer family functioning at T1 predicted higher child anxiety t T2 and poorer family functioning at T2 predicted higher child depressive symptoms at T3.

## Managing emotions

Figure 4 shows the associations between *managing emotions *and child anxiety and depressive symptoms. There was a statistically significant, negative correlation between *managing emotions *and child depression at T3. Parents fostering emotion regulation and resilience was thus associated with lower child depressive symptoms one year after the intervention.

## Setting goals & dealing with problems

Figure 5 shows the associations between *setting goals & dealing with problems* and child anxiety and depressive symptoms. There were statistically significant, negative correlations between *setting goals & dealing with problems* and child anxiety and depression at T2, and child anxiety at T3. Helping children set realistic goals, solve problems and persevere through challenges was associated with lower anxiety and depressive symptoms at T2, and anxiety levels one year later.

## Dealing with negative emotions

Figure 6 shows the associations between *dealing with negative emotions* and child anxiety and depressive symptoms. There were statistically significant, positive correlations between *dealing with negative emotions* and child anxiety and depressive symptoms at T1. Parents’ support in helping children manage and cope with their emotions was associated with higher anxiety and depression scores. By T2, these associations were negative, parents’ support was associated with lower levels of child anxiety and depression.

## Discussion

This study examined the relationship between parent-reported child anxiety and depressive symptoms and parental factors related to child anxiety and depression: parents’ anxiety and depressive symptoms, family functioning, and three parental practices. The fit between our hypothesized models and the observed data was relatively good.

Parents’ symptoms correlated positively with child anxiety and depression at all three time points, indicating mutual reinforcement of symptoms. This aligns with previous research [2, 3] identifying parental symptomatology as a risk factor for anxiety and depression. Additionally, parents’ symptoms at baseline and post-intervention predicted child depressive symptoms at the later measurement time. One possible explanation is that children of more symptomatic parents have poorer intervention outcomes, as shown in Eckshtain et al. [4]. This suggests that anxious or depressed parents might be less able to support and reinforce the effects of the *Emotion* program post-intervention. However, the analyses in the current study does not provide evidence of how parental factors related to child anxiety and depression affected children’s treatment outcomes, and this should be examined in another study.

Poorer family functioning correlated with child anxiety and depression at all three measurement times. The FAD-GF scale evaluates communication patterns, emotional bonds and acceptance levels, and problem-solving abilities. Our results suggest that poorer family functioning may lead to increased anxiety and depression in children, and vice versa, in line with the triadic model [1]. Children with higher symptom levels of anxiety and/or depression can negatively impact family functioning through disengaged behavior and poor communication. Baseline family functioning predicted child anxiety post-intervention, indicating that children from families with poorer family functioning have higher levels of anxiety after the *Emotion* intervention compared to those from better functioning families. Additionally, post-intervention family functioning predicted child depression one year later, suggesting that a supportive, low-conflict family environment can help reduce child depression in the time following an intervention. These results align with the universal prevention study by Kennedy et al. [32], where children’s perceived family functioning at baseline predicted child internalizing symptoms six months later. The authors suggest that baseline family functioning may moderate intervention effects on child anxiety and depression, with children from poorer-functioning families benefiting less [32]. It is reasonable to believe that poor family functioning, characterized by low levels of support, poor communication, and high conflict levels may hinder parents from effectively implementing intervention strategies, such as behavioral experiments. These results support the evidence that poor family functioning is a risk factor for child emotional problems and should be screened for, to identify children who might need additional support. The *Emotion* intervention encourages parents to set aside time for positive interactions with their children and support them during difficult situations. Results indicate that future interventions or versions of the *Emotion* intervention could focus more on improving overall family functioning, particularly through better communication.

To the best of our knowledge, this is the first study to examine the association between specific parental practices and child anxiety and depression in an indicated preventive intervention study. The associations between child anxiety and depression and the included parental practices were smaller than with parent symptomatology and family functioning. This could be expected, as parental practices perhaps vary more over time and situations than parents´ mental health or overall family functioning. Sim et al. [12] found small, negative correlations between the total PaRCADS score and children’s anxiety and depressive symptoms, indicating that more adaptive parenting predicted lower symptom scores. However, a Norwegian study did not replicate these findings [25]. In this study, all correlations between child symptoms and *managing emotions* and *setting goals & dealing with problems* were negative, except for child anxiety at baseline. Although non-significant, the direction of these associations, suggests proficient parental support in managing emotions, setting clear and realistic goals, and improving children’s problem-solving-skills, may reduce child anxiety and depression. The current sample consists mainly of affluent, well-educated parents with comparable PaRCADS(N) scores to those of a community sample [25]. Conducting these analyses on a sample with less adaptive parental practices might yield stronger, or different outcomes.

There was a statistically significant negative correlation between *dealing with* emotions and child depression at 12-month follow-up. A recent study [18] on the same sample, showed that parental practice *dealing with negative emotions* improved from baseline to post-intervention and remained stable until 12-month follow-up. This suggests that an improvement in how parents foster emotional intelligence, resilience, and a balanced perspective in their child, during the *Emotion* intervention, may have led to further reduction in symptoms over the following year. It is also plausible that an improved ability to support their child in managing negative emotions has led to changes in parents’ perceptions and reports of child symptoms.

Post-intervention, there were statistically significant, negative associations between *setting goals & dealing with problems* and child anxiety and depression. In Ytreland et al. [18], using data from the same trial, parents’ scores in this domain increased from baseline to post-intervention but returned to baseline levels after 12 months. During the initial sessions, children set SMART goals (Specific, Measurable, Achievable, Relevant, and Time-Bound) with group leaders and parents, which were monitored throughout the intervention. Their results suggest that parents improved their ability to foster resilience and problem-solving skills in their children, at least short term [18], which may have contributed to decreased symptoms in their children. Only the correlation with anxiety remained stable one year later, indicating that parents’ role in helping their children set goals, persevere and deal with problems may be more important to reduce child anxiety than depression.

There were positive correlations between *dealing with negative emotions* and child anxiety and depression scores at baseline, suggesting that the more parents focused on negative emotions, the more anxious and depressive symptoms their children had. Interestingly, this correlation shifted from positive to negative for both anxiety and depression post-intervention. While the *Emotion* intervention reduced children’s anxiety and depressive symptoms [17], it also aimed to equip parents with strategies to help their children develop emotional resilience, self-regulation, and coping skills. This may have improved parents’ understanding of their children’s anxious and depressive thoughts. Also, parents who are more attentive to their children’s negative emotions may rate their children as more anxious and/or depressive due to an attention bias.

The findings from Lisøy et al. [17] demonstrate promising effects of the intervention on child anxiety and depression symptoms, irrespective of the parental involvement format (i.e., psychoeducational brochure or five parent sessions). Further, Ytreland et al. [18] found no significant between-group differences in parental factors related to child outcomes. These findings suggest that even minimal parental involvement (a brochure and one information meeting), may be sufficient for intervention success. These findings highlight the potential of *Emotion* as a low-intensity, school-based intervention for addressing internalizing symptoms in children. The original parental component of *Emotion* (five group sessions) does not appear to enhance outcomes for children’s anxiety or depression when compared to a less resource-intensive psychoeducational brochure. This suggests two possible courses of action in clinical practice: enhancing the parent group sessions of the intervention to improve its effectiveness, or removing them from the intervention altogether.

According to Schleider & Weisz’ model [1], optimizing parental factors on the different levels, i.e. parents’ anxiety and depressive symptoms (parent-level), family functioning (family-level) and parental practices (dyad-level), may help initiate a positive spiral. This could lead to sustained improvements in child outcomes beyond the duration of the intervention, which is supported by the associations between parental factors and children’s symptoms in the current study. Future research should explore the impact of a preventive intervention component that targets these maintaining parental factors, positioning parents as the main client, rather than facilitators for their children. One example is the universal, online-based *PaRK*-intervention, which focuses on parental risk and protective factors for child anxiety and depression [11]. While it has shown effectiveness in improving relevant parental practices, no mediating effects on children’s symptoms were observed one-year post-intervention [11]. It would be valuable to investigate whether different results emerge in samples with higher baseline symptom levels, or at later follow-up points.

If the parent group sessions were to be discontinued, *Emotion* would consist of sixteen children’s sessions and a parent brochure together with one informational parent meeting. This could be well suited for integration into stepped care models, which prioritize delivering the least intensive, yet effective, interventions first, reserving more intensive approaches for those with greater need [33]. Implementing *Emotion* within such a framework could help close the treatment gap for internalizing problems in children by offering early, accessible support in schools or other low-intensity settings. Its feasibility with minimal demands on parents offers both clinical and logistical advantages, particularly in contexts where time, resources, or parental engagement are limited. Future studies should examine the long-term sustainability of outcomes under stepped care conditions and identify which children may benefit from additional parental components.

## Strengths and limitations

The ECHO trial involved 701 children in the intervention nationwide with a low dropout rate of 6% [17]. The sample included both sexes and varied in terms of both parents’ and children’s anxiety and depression levels. Trained professionals from first-line services led the intervention in real-life settings, enhancing generalizability and ecological validity, and the current study contributes valuable knowledge as research on parental factors related to child anxiety and depression in preventive settings has been limited.

There are also limitations in this study. Data was available from 80% of parents for 93% of children. However, parent response rates declined over time: from 72% at baseline to 42% at 12-month follow-up, corresponding to 88%, and 63% of the children, respectively. The sample primarily included educated, employed parents born in Norway with a stable household economy, which potentially limits the generalizability of our results. Further, some findings are correlational by nature and may be influenced by common source variance, preventing causal conclusions.

Analyses relied solely on parent-reported data, which can be both a strength and a weakness. Parents provide a broad perspective, but may be biased, while children offer direct insights into their inner world, but may be influenced by immediate mood or recent events [34]. Optimally we would include observations of the parental factors and children´s symptoms. However, surveys are a reasonable alternative given the size of this trial.

Domains from PaRCADS(N) had lower internal consistency values, consistent with previous research, there is little research on this norm-referenced version of PaRCADS, and these results should be interpreted with care.

It would be interesting to explore whether boys and girls, who were included based on their anxiety or depression (or both) scores, are affected differently by parental factors, and if parents with higher symptom levels affect their children’s symptoms differently. Future research should also examine how the parental factors interact.

## Conclusion

This study enhances our understanding of the associations between child anxiety and depression and parental factors such as anxiety and depressive symptoms, family functioning, and parental practices, before, immediately after, and one year after a preventive intervention. Our results indicate that children’s anxiety and depressive symptoms are more strongly associated with parents’ anxiety and depressive symptoms and family functioning than with parental practices. Future research should explore whether emphasizing relevant parental factors within preventive interventions can lead to improved outcomes for children.

## Data Availability

Data is not publicly available due to privacy restrictions, but can be made available upon reasonable request to senior authors.
